# Unravelling the multi-target mechanism of methoxylated flavonoids in Parkinson's disease: Insights from network pharmacology and molecular dynamics

**DOI:** 10.1016/j.toxrep.2026.102248

**Published:** 2026-04-01

**Authors:** Chetan Ashok, Srikanth Jeyabalan, Parasuraman Pavadai, Vignesh M, Devaraja Dravida Pandiyan S, Durga Mohan, Revathi Sugumar, Ramya Sugumar, Sugin Lal Jabaris, Vetriselvan Subramaniyan, Ling Shing Wong, Mahendran Sekar

**Affiliations:** a Department of Pharmacology, Faculty of Pharmacy, Sri Ramachandra Institute of Higher Education and Research (DU), Chennai, Tamil Nadu 600116, India; b Department of Pharmaceutical Chemistry, Faculty of Pharmacy, M.S. Ramaiah University of Applied Sciences, Bengaluru, Karnataka 560054, India; c Department of Pharmaceutical Regulatory Affairs and Management, Manipal College of Pharmaceutical Sciences, Manipal Academy of Higher Education, Manipal, Karnataka 576104, India; d Department of Pharmacology, Sri Ramachandra Medical College, Sri Ramachandra Institute of Higher Education and Research (DU), Chennai, Tamil Nadu 600116, India; e Department of Pharmacology, Siddha Central Research Institute, Central Council for Research in Siddha, Anna Hospital Campus, Arumbakkam, Chennai 600106, India; f Department of Biomedical Sciences, Sir Jeffrey Cheah Sunway Medical School, Faculty of Medical and Life Sciences, Sunway University, Petaling Jaya, Selangor Darul Ehsan 47500, Malaysia; g Faculty of Health and Life Sciences, INTI International University, Putra Nilai, Nilai, Negeri Sembilan 71800, Malaysia; h School of Pharmacy, Monash University Malaysia, Bandar Sunway, Subang Jaya, Selangor, Malaysia

**Keywords:** Methoxylated flavonoids, Network pharmacology, Parkinson’s disease, Molecular dynamics, Molecular docking, Mental health

## Abstract

Current therapy for Parkinson's disease (PD), a neurodegenerative disorder that progresses over time marked by protein aggregation, dopaminergic neuronal death and oxidative stress, only provides symptomatic alleviation. By using an integrative computational strategy that combines molecular dynamics simulations, molecular docking and network pharmacology, this study sought for the investigation of the neuroprotective potential of methoxylated flavonoids (MFs). Drug-likeness and ADMET profiling of ten MFs revealed favorable pharmacokinetics, including good oral bioavailability and low predicted toxicity. Network pharmacology identified 172 putative targets, of which 118 overlapped with PD-associated genes, highlighting the multi-targeted nature of these compounds. Protein–protein interaction analysis revealed important hub proteins like HSP90AA1, MMP9, GSK-3β and AKT1 involved in neurodegeneration, oxidative stress regulation, and apoptotic signaling. Analysis of KEGG and GO pathway enrichment revealed significant modulation of the signaling pathway PI3K-Akt and the cellular elements necessary for neuronal survival and repair. Molecular docking revealed high-affinity interactions of Eupatilin and Norwogonin with key PD targets, particularly HSP90AA1 and MMP9. Molecular dynamics simulations confirmed the Eupatilin–HSP90AA1 complex's stability over a 100 ns period, indicating stable interactions and minimal conformational fluctuations. These findings suggest that methoxylated flavonoids possess multi-target therapeutic potential against PD, contributing to broader goals in public health and mental health by modulating interconnected neuroprotective pathways. However, as the results are predictive in nature, confirming their translational value requires experimental confirmation through *in vitro* and *in vivo* research. Study highlights Eupatilin as a potential lead substance for additional pharmacological research aimed at disease modification in Parkinson’s disease.

## Highlights

• Methoxy flavonoids show multi-target neuroprotective potential against PD.
• Network analysis identified 118 shared targets, highlighting HSP90AA1 and MMP9.
• Flavonoids modulate PI3K-Akt signaling, apoptosis, and oxidative stress in PD.
• Eupatilin shows strong, stable binding to HSP90AA1 in 100 ns MD simulations.
• These compounds hold promise as multi-target agents for modifying PD progression.

## Graphical abstract

**Figure:**
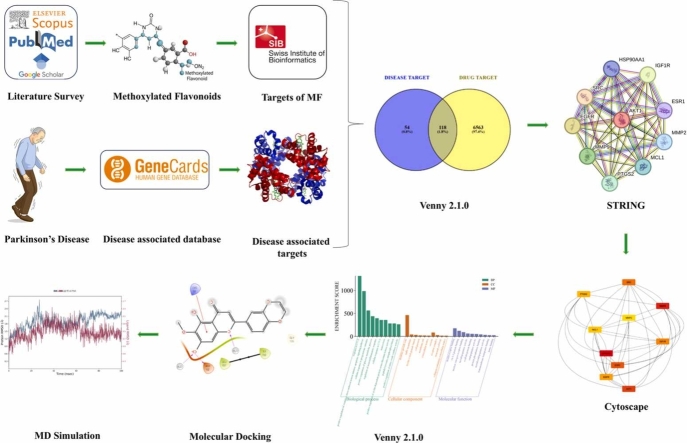

## 1 Introduction

Neurodegenerative diseases are characterized by the progressive decline and loss of function of brain neurons, which ultimately results in impaired neuronal communication and disrupted brain activity. As these conditions advance, they manifest through clinical symptoms such as muscle weakness, impaired coordination, and a decline in overall physiological function. The progressive nature of NDDs severely compromises the standard of living of an individual often hindering the capability of carrying out habitual everyday tasks and maintain functional independence [1], [2]. In neurodegenerative disorders, post-mitotic nerve cells progressively lose their functional integrity and undergo cell death, predominantly through apoptosis, a controlled form of programmed cell death. Apoptosis contributes to disease pathology by disrupting intracellular homeostasis and amplifying cellular damage. This process is further exacerbated by oxidative stress, a state marked by the overaccumulation of ROS beyond the capacity of antioxidant protection in the body systems, resulting in further neuronal injury and dysfunction [3]. In addition to neuronal loss, NDDs are propelled by a complex interplay of molecular, genetic, and biochemical factors. that collectively exacerbate neuronal dysfunction and degeneration. Emerging research has identified the presence of abnormal protein species in the brains of individuals with NDDs. These proteins, which normally play essential roles in cellular homeostasis, undergo misfolding, aggregation, or pathological modifications. Such aberrant protein conformations disrupt normal cellular functions and lead to toxic accumulation within neurons. This proteinopathy not only disturbs cellular equilibrium but also activates pathological processes that accelerate neuronal damage and disease progression [4].

Parkinson’s disease ranks among the most frequent neurodegenerative disorders, mainly impacting middle-aged and older adults [5]. Its global incidence continues to rise, driven by aging populations, improved diagnostic capabilities, and increased public awareness. Despite extensive research efforts, PD remains incurable, and current treatment strategies are limited to symptomatic relief and enhancing patients' life quality. The increasing worldwide load of PD highlights the urgent need for ongoing research aimed at elucidating its underlying pathophysiological mechanisms and developing novel therapeutic interventions [6], [7]. It is a progressively deteriorating neurodegenerative disorder characterized by a combination of motor and non-motor symptoms that significantly impair quality of life. Motor manifestations include resting tremors, muscular rigidity, bradykinesia (slowness of movement), gait disturbances such as shuffling or freezing, and postural instability, which heightens the risk of falls. In addition, behavioural disturbances including apathy and emotional dysregulation are frequently observed. Symptoms that are not motor-related, including cognitive decline, sleep disturbances, mood changes, olfactory dysfunction, anxiety and depression further contribute to the disease burden. Emerging evidence suggests that neuroinflammation and the accumulation of cytotoxic mediators, including interleukin-1 (IL-1), nitric oxide, Tumor necrosis factor and reactive oxygen species play critical roles in exacerbating neuronal damage and compromising neural integrity in PD [8], [9], [10], [11].

Sleep disorders (SDs) represent among the frequently observed non-motor manifestations associated with Parkinson’s disease, impacting around 60–90% of patients and causing significant disruption to the sleep-wake cycle. People with PD commonly experience sleep disturbances like insomnia, sleep behaviour disorder (where individuals act out dreams), REM, excessive daytime sleepiness, restless legs syndrome and fragmented sleep. These sleep disturbances exacerbate fatigue, cognitive decline, and mood disorders, thereby increasing the overall disease burden. Effective management of sleep disorders is essential for overall health and welfare and enhancing the standard of living for PD patients [12].

Parkinson’s disease is primarily managed using pharmacological agents that restore or mimic dopaminergic activity. The cornerstone of treatment is levodopa, typically administered in combination with carbidopa to inhibit peripheral degradation and enhance central nervous system availability. Additional therapeutic options include anticholinergic agents, catechol-O-methyltransferase inhibitors, monoamine oxidase-B inhibitors, dopamine agonists and amantadine. Although these medications alleviate motor symptoms, they are frequently linked to adverse effects like nausea, hallucinations and dyskinesia and do not halt the underlying neurodegenerative process. These limitations highlight the critical need for the advancement of new treatment strategies that not only minimize side effects but also modify disease progression to improve long-term outcomes in PD patients [13], [14], [15].

**Fig. 1:**
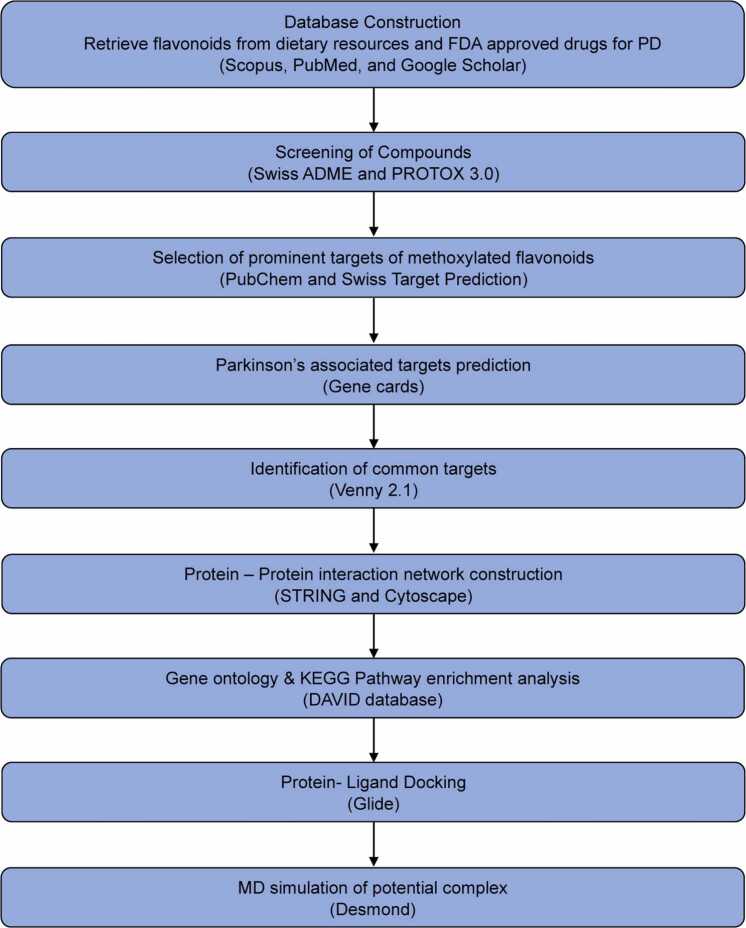
Schematic illustration of the study’s complete workflow.

Network pharmacology employs computational biology, systems biology, and other cutting-edge technologies to explore the relationships between phytochemical treatments and diseases [16], [17]. It offers an entire view of drug action that transcends the conventional method of "one drug, one target" and emphasizes the coordination of multiple targets [18]. Molecular docking represents a computational methodology in bioinformatics that leverages the properties of receptors and their interactions with pharmaceutical compounds for drug design. Its focus lies in the investigation of the binding patterns and affinities that exist between ligands and receptors. On the other hand, molecular dynamics simulation investigates the dynamic behavior of biological macromolecules such as proteins and DNA. This method simulates their movement in a three-dimensional space, uncovering the mechanisms of biological functions and assisting in identifying small molecules and potential targets [19], [20], [21]. Within this research, the authors used molecular docking, network pharmacology and molecular dynamics simulation to explore pathways, mechanisms of action, and potential targets of methoxylated flavonoids (MFs) in managing PD, thereby establishing a basis for informed clinical application of treatment. Fig. 1 shows the workflow of the study.

## 2 Materials and methods

This experimental study includes 9 phases: Database construction, screening of compounds, a selection of prominent targets of Methoxylated flavonoids, Parkinson’s associated targets prediction, identification of common targets of PD, creation of protein-protein interaction network, enrichment analysis, molecular docking for protein-ligand interactions, and molecular dynamic simulation.

### 2.1 Database construction (search strategy)

It has always been recognized that natural polyphenols can have positive effects on a range of diseases. A comprehensive literature search conducted with various search engines like Google Scholar, PubMed, and Scopus using keywords like, “PD and methoxylated flavonoids,” “antioxidants and PD” and “and antioxidants, FDA-approved drugs and Parkinson’s disease” ([22]), the following compounds including Nobiletin, Nevadensin, 3,5,6,7,8-Pentamethoxyflavone, Eupatilin, Butein, Norwogonin, Tricin, Wogonin, 5-Hydroxy-3,7,8,3′,4′-pentamethoxyflavone and 6-Methoxytricin appeared as search results. These 10 compounds (In supplementary file S1 you can see the structures of the ligands) have been noted for their association with anti-aging effects and neuroprotective effect. They are advantageous in combating neurodegenerative diseases comprising Alzheimer’s disease [23], [24], [25], [26], [27], [28], [29], [30], [31], [32], [33], [34], [35]. Additionally, chosen ligands were utilized to create dataset and to forecast their interacting partners and biomolecular targets.

### 2.2 Screening of compounds

#### 2.2.1 Drug-likeliness prediction

The selected 10 MFs canonical smiles (represented in Table 1) along with two reference drugs, levodopa and 17-DMAG, were obtained from the PubChem database (https://pubchem.ncbi.nlm.nih.gov/, obtained on October 9, 2024), online database, and entered into the SwissADME web server (http://www.swissadme.ch/, online database, accessed on October 9, 2024) to assess their drug-likeliness [36]. The drug-like characteristics of the MFs were assessed using several parameters like topological polar surface area, bioavailability score, and lead-likeness which determine the gastrointestinal absorption and brain permeability of the chosen ligands [37], iLOGP, ESOL class and ESOL Log S (water solubility) and the Lipinski’s rule of five (molecular weight must be under 500 Dalton (500 g/mol), the quantity of hydrogen bond donors should be under five, lipophilicity should be high, molar refractivity should fall within (40–130) and fewer than ten hydrogen bond acceptors [38]. It is required that the bioavailability (F) value exceeds 30%, with F30 indicating a 30% bioavailability. The bioavailability of a medicinal product refers to the speed and extent to which its active components are absorbed into the blood circulation. ADME characteristics exemplify the drug-likeness. On Boiled Egg graphical interface, with WLogP and TPSA values depicted on the X and Y axis, in the same order. TPSA represents the ligand's accessibility, while WLogP denotes its lipophilicity. The active component was the one with a bioavailability score exceeding 0.5 [39].

**Table 1:** Drug-likeness prediction using SwissADME**.**

**S.NO**	**Methoxylated flavonoids**	**PUBCHEM ID**	**Molecular formula**	**Molecular weight (g/mol)**	**TPSA**	**#H-bond acceptors**	**#H-bond donors**	**WLOGP**	**MLOGP**	**MR**	**#Heavy atoms**	**GI absorption**	**Bioavailability Score**	**Lipinski #violations**	**Ghose #violations**
1	Levodopa	6047	C9H11NO4	197.19	103.78	5	4	0.05	-2.26	49.55	14	High	0.55	0	0
2	Nobiletin	72344	C21H22O8	402.39	85.59	8	0	3.51	0.34	106.87	29	High	0.55	0	0
3	Nevadensin	160921	C18H16O7	344.32	98.36	7	2	2.9	0.17	91.44	25	High	0.55	0	0
4	3,5,6,7,8-Pentamethoxyflavone	471720	C20H20O7	372.37	76.36	7	0	3.5	0.63	100.38	27	High	0.55	0	0
5	Eupatilin	5273755	C18H16O7	344.32	98.36	7	2	2.9	0.17	91.44	25	High	0.55	0	0
6	Butein	5281222	C15H12O5	272.25	97.99	5	4	2.3	1.02	74.34	20	High	0.55	0	0
7	Norwogonin	5281674	C15H10O5	270.24	90.9	5	3	2.58	0.52	73.99	20	High	0.55	0	0
8	Tricin	5281702	C17H14O7	330.29	109.36	7	3	2.59	-0.07	86.97	24	High	0.55	0	0
9	Wogonin	5281703	C16H12O5	284.26	79.9	5	2	2.88	0.77	78.46	21	High	0.55	0	0
10	17-DMAG	5288674	C32H48N4O8	616.75	169.52	9	4	1.53	-0.72	170.07	44	Low	0.17	2	3
11	5-Hydroxy-3,7,8,3′,4′-pentamethoxyflavone	10200272	C20H20O8	388.37	96.59	8	1	3.21	0.11	102.4	28	High	0.55	0	0
12	6-Methoxytricin	14034284	C18H16O8	360.31	118.59	8	3	2.6	-0.35	93.46	26	High	0.55	0	0

#### 2.2.2 ADMET profiling

By using the web server ProTox 3.0 (https://tox.charite.de/protox3/, online database, accessed on October 9, 2024) the toxicity characteristics of the 10 ligands along with reference drugs was assessed [40]. The corresponding ligands' SMILES were integrated into the web server for analysis and to estimate compound’s LD50 value. The substances were distributed among classes 1–6 according to the nature of their toxicity. Class 1 denotes that the compound is highly toxic, while Class 6 denotes that the compound is non-toxic.

A Class 4 toxicity limit was established as the primary filter for compound selection in this study [41]. Furthermore, the pkCSM web server (https://biosig.unimelb.edu.au/pkcsm/, accessed on October 10, 2024) was utilized to comprehensively evaluate the pharmacokinetic profiles of the ligands. This evaluation assessed key ADMET parameters, including water solubility, intestinal absorption, Caco-2 permeability, skin permeability, blood-brain barrier (BBB) and CNS permeability, volume of distribution, enzyme interactions, P-glycoprotein inhibition, and renal clearance. This assessment aimed to filter potential compounds against PD [42], [43].

### 2.3 Selection of prominent targets of methoxylated flavonoids

From the PubChem database the structures of MFs were obtained, with the analysis specifically tailored to human biological targets through selecting "Homo sapiens" as the species. Canonical SMILES of these compounds were processed with a probability cut-off of > 0 using the Swiss Target Prediction website (http://www.swisstargetprediction.ch/, 2023 version, accessed on October 10, 2024) to retain all likely predicted interactions. To make sure that no potentially relevant interactions were omitted at the outset, the deliberately chosen inclusive threshold allowed for a broad preliminary mapping of the flavonoid–target space. While an inclusive threshold was used for initial target harvesting, the subsequent intersection with the highly restricted Parkinson's disease gene dataset (GeneCards relevance score ≥15) served as the primary, stringent biological filter to eliminate false positives. Applying a stricter initial probability threshold (e.g., >0.15) would reduce the overlapping target count to 47, risking the exclusion of biologically relevant secondary nodes. This tool uses 2D and 3D similarity-based algorithms to find possible human protein targets, enabling exploration of pharmacological interactions and the bioactivity of methoxy flavonoid compounds [44].

### 2.4 Parkinson’s associated targets prediction

Using the GeneCards database (https://www.genecards.org/, obtained on October 10, 2024, version 5.12) genes associated with Parkinson's disease were identified a comprehensive platform integrating genomic, proteomic, and clinical data. A search with the keyword "Parkinson's disease" generated a ranked list of genes based on relevance scores threshold of ≥ 15. In network pharmacology studies this score is based on high-confidence associations derived from transcriptomic, integrated genomic, proteomic evidence, and aligns with practices commonly adopted. These strict criteria contributed to an increase in disease specificity and a reduction in the inclusion of weak or indirect associations. Specifically, utilizing a lower threshold (<15) would artificially inflate the absolute number of overlapping targets by introducing significant noise and false positives into the network. Conversely, applying a stricter threshold (e.g., ≥30) would excessively restrict the disease gene pool, risking the omission of valid secondary targets essential for capturing the multi-targeted pharmacological nature of methoxylated flavonoids. This analysis provided insights into Parkinson’s pathogenesis and helped identify potential therapeutic targets for developing tailored treatments [45].

### 2.5 Identification of common targets

In order to determine shared therapeutic targets, (using Venny 2.1.0) Venny diagram was created (accessed on October 12, 2024, https://bioinfogp.cnb.csic.es/tools/venny/) through a comparison of the anticipated pharmacological targets of methoxylated flavonoids with genes associated with PD [46].

### 2.6 Protein – protein interaction network construction

The STRING database (https://string-db.org/, version 12, accessed on October 12, 2024) was used to generate a protein-protein interaction network aimed at investigating functional interactions that play a role in chromone's anti-Parkinson effects. To find relevant targets, using the Cytoscape software (version 3.10.2) network was analyzed confidence score (≥0.4) and "Homo sapiens" as the selection [45], [47], [48].

#### 2.6.1 Construction of compound targets network

In network pharmacology, Cytoscape software is essential for observing and analysing biomolecular interaction networks. It creates compound-target networks for methoxy flavonoids using criteria such as pharmacological similarity and bioavailability. Predicted targets from Swiss Target Prediction are compared against targets linked to Parkinson's disease from the GeneCards database and any overlap is presented in Cytoscape (accessed on October 13, 2024: Version 3.10.2,). Topological parameters, including average degree, network diameter, and density, were calculated to evaluate the connectivity and complexity of the network. Hub genes were subsequently identified using the CytoHubba plugin with the Maximal Clique Centrality (MCC) score to ensure robust detection of key targets. MCC was specifically selected as the primary ranking metric because it is widely recognized for its superior accuracy and robustness in predicting essential proteins and identifying functional core modules within complex networks compared to standard centrality measures. Cytoscape's tools, together with plugins such as EnrichmentMap, examine important metabolic pathways, demonstrating how methoxy flavonoids influence these processes. Its extensive visualization capabilities give a clear picture of compound-target-pathway networks, helping to make sense of complex interactions and improving knowledge of the therapeutic benefits of methoxy flavonoids in PD [49].

### 2.7 Gene ontology & KEGG pathway enrichment analysis

GO and KEGG pathway analyses were performed to clarify the potential neuroprotective mechanisms of methoxy flavonoids in PD. Target genes linked to methoxy flavonoids were identified through bioinformatics databases, and their functional annotations were systematically retrieved. The Database for Annotation, Visualization and Integrated Discovery (DAVID) (https://davidbioinformatics.nih.gov/, version 2021, accessed on October 14, 2024) was employed to categorize these genes into Gene Ontology domains:, Molecular Functions, Cellular Components, Biological Processes. To control for false positives across multiple comparisons, p-values were corrected using the False Discovery Rate (FDR) adjustment (Benjamini-Hochberg method), with an FDR-adjusted p-value < 0.05 deemed as statistically significant. Additionally, to identify relevant signaling pathways, KEGG pathway analysis was performed. using the DAVID database, enrichment analysis of the overlapping hub genes was conducted, with the species restricted to Homo sapiens, offering perspectives on the biological functions and pathways potentially modulated by methoxy flavonoids [50], [51], [52] and the outcomes were illustrated graphically with SRplot (https://www.bioinformatics.com.cn/en, web platform, accessed on October 14, 2024)

### 2.8 Molecular docking

Molecular docking attempts to identify each ligand's binding partners by enabling the examination of ligand-protein interactions. Schrödinger Suite from the Protein Preparation Wizard was utilized for protein structure preparation (Academic version 2023–1, accessed on October 20, 2024). During this process, Water molecules that were not essential were eliminated, and the structures were fine-tuned with the OPLS4 force field resulting in a RMSD of 0.30 Å. Hydrogen bond optimization had been carried out using the PROPKA module. Subsequently, by using the Receptor Grid Generation tool the receptor grid was created and defines the active site for docking studies. In this study, MFs were docked with two target proteins, Heat Shock Protein 90 (HSP90AA1; PDB ID: 7LT0; resolution: 1.70 Å) and Matrix Metalloproteinase-9 (MMP9; PDB ID: 8K5Y; resolution: 1.52 Å).

These specific targets were prioritized from the larger pool of hub genes to strategically evaluate two distinct, critical hallmarks of Parkinson's disease pathology: protein misfolding and α-synuclein aggregation (HSP90AA1), and neuroinflammation associated with blood-brain barrier disruption (MMP9). The Extra Precision (XP) docking protocol was employed to perform molecular docking by using the Glide a component of the Schrödinger Suite estimates how strongly compounds bind to their respective protein targets. Protein-ligand interactions, including hydrophobic and hydrogen bonding interactions, and binding scores (in kcal/mol) were closely analysed to determine their potential therapeutic value ([53],[54]).

### 2.9 Molecular dynamics simulations

Molecular dynamics simulations were conducted using Schrodinger’s Desmond software (Academic version 2023–1), simulation performed on November 21, 2024) using the OPLS 2005 force field for a duration of 100 ns to examine the stability and from molecular docking, dynamic behaviour of the protein-ligand complex obtained. Due to computational demands, this 100 ns simulation was exclusively reserved for the top lead candidate, Eupatilin, while reference compounds served as baselines for the earlier docking and ADMET evaluations. The complex was prepared by removing unwanted chains, water molecules, and heteroatoms, then solvated within an orthorhombic box of simple point charge water molecules subject to periodic boundary conditions to neutralize the system, (Cl- or Na+) counterions were introduced, following which energy minimization was performed. Under NPT ensemble conditions (300 K and 1.01325 bar), the system was equilibrated and underwent a 100 ns production run using a 2-fs time step. The root mean square deviation (RMSD), root mean square fluctuation (RMSF), and radius of gyration (Rg) were estimated to determine stability and dynamic behavior. RMSD values remained consistent (under 2.5 Å), and Rg was stable, indicating that structural integrity was maintained and ligand binding was appropriate. Hydrogen bonding and hydrophobic interactions confirmed the stability of the complex throughout the simulation [55], [56].

## 3 Results

Levodopa, a clinically approved drug for PD, and 17-DMAG (Alvespimycin), a well-established inhibitor of both HSP90AA1 and MMP9, were utilized as reference compounds for target validation. Network pharmacology analysis was subsequently carried out for MFs to investigate their possible therapeutic function in the management of PD.

### 3.1 Screening of compounds

#### 3.1.1 Drug-likeliness prediction

**Fig. 2:**
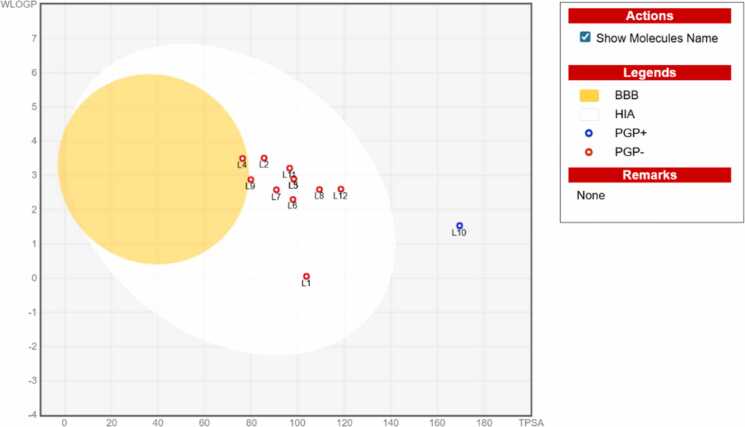
Graphical representation of 12 ligands as a boiled egg using SwissADME. L1-L12 denote ligands 1–12 as listed in Table 1.

Lipinski’s rule of five and the Ghosh filter were applied to evaluate the drug-likeness of the ligands, as predicted by Swiss ADME. For each ligand, drug-likeness parameters, including logP, MW, the count of WLOGP, the count of hydrogen-bond donors, MLOGP, hydrogen-bond acceptors, MR and the atom count, were predicted. Each compound satisfied the Ghose and Lipinski filter requirements, confirming their drug-like nature without any infringements. The reference medication, 17-DMAG, demonstrated breaches of the Lipinski and Ghose filters. Table 1 illustrates the results regarding the drug-likeness of each ligand. The BOILED-Egg graphical model (Fig. 2) predicted that 3,5,6,7,8-pentamethoxyflavone possesses a high probability of crossing the blood-brain barrier. In contrast, the remaining methoxylated flavonoids, along with the reference drug 17-DMAG, exhibited lower probabilities of BBB permeation and were classified as non-brain penetrant [36], [57].

#### 3.1.2 ADMET profiling

The toxicity profile for all 10 flavonoids and 2 reference drugs was evaluated using the ProTox 3.0 web server (shown in Table 2). The results indicated that, apart from 17-DMAG, the other ligands exhibited high LD50 values. Discrepancies were observed between BBB permeability predictions from ProTox-3.0 and the SwissADME boiled egg plot. These variations stem from the different predictive algorithms and training datasets utilized by each platform, such as the physicochemical models in SwissADME versus the machine learning and fragment-based models in ProTox-3.0. To mitigate this algorithmic bias and obtain a more robust, quantitative assessment, the pkCSM web server was employed to conduct an extensive examination of the compounds' comprehensive ADMET properties.

**Table 2:** Toxicity results using ProTox – 3.0 Software. BBB: Blood Brain Barrier; LD50: Median Lethal Dose.

**LIGANDS**	**LD50 Value (mg/kg)**	**Toxicity class**	**Hepatotoxicity**	**Neurotoxicity**	**Carcinogenicity**	**Immunotoxicity**	**Mutagenicity**	**Cytotoxicity**	**BBB-barrier**
Levodopa	1460	4	Inactive	Inactive	Inactive	Inactive	Active	Inactive	Inactive
Nobiletin	5000	5	Inactive	Inactive	Inactive	Active	Inactive	Inactive	Active
Nevadensin	3919	5	Inactive	Inactive	Inactive	Active	Inactive	Inactive	Inactive
3,5,6,7,8-Pentamethoxyflavone	5000	5	Inactive	Inactive	Inactive	Inactive	Inactive	Inactive	Active
Eupatilin	5000	5	Inactive	Inactive	Inactive	Active	Inactive	Inactive	Inactive
Butein	1000	4	Inactive	Inactive	Active	Active	Inactive	Inactive	Active
Norwogonin	3919	5	Inactive	Inactive	Active	Inactive	Active	Inactive	Active
Tricin	4000	5	Inactive	Inactive	Inactive	Inactive	Inactive	Inactive	Inactive
Wogonin	3919	5	Inactive	Inactive	Inactive	Inactive	Inactive	Inactive	Active
17-DMAG	27	2	Inactive	Inactive	Inactive	Active	Inactive	Inactive	Inactive
5-Hydroxy-3,7,8,3′,4′-pentamethoxyflavone	5000	5	Inactive	Inactive	Inactive	Active	Inactive	Inactive	Active
6-Methoxytricin	1190	4	Active	Active	Inactive	Active	Inactive	Inactive	Inactive

Water solubility values predicted by the pkCSM web server typically range from −6 to 0, where lower values indicate poor aqueous solubility, potentially leading to challenges in absorption and formulation. Caco-2 permeability values ranging from −1 to 1.5 suggest that the compound possesses adequate permeability across the intestinal epithelium. A potential for high oral bioavailability and Good permeability are indicated by a higher value, while poor absorption is suggested by a lower value. Intestinal absorption varies from 0 to 100. Skin permeability is valued between − 3 and − 1.Compounds with values exceeding 80% are considered to have high absorption. Values that are lower suggest that the compounds have low skin permeability, which points to their significance for transdermal or topical drug delivery. The property of inhibiting P-glycoprotein suggests that the compound may influence drug efflux and raise the concentration of drugs within cells. The distribution volume value varies from −2 to 2.

A greater value signifies that the compound is widely distributed across the tissues, while a lesser value shows that the compound is confined to the plasma. The value of BBB permeability is in the midst of −3 and 1. Values that are close to 0 or positive suggest good BBB permeability. Moreover, CNS permeability varies from −4 to 0. Values that are higher show better penetration, which is crucial for brain-targeting drugs. The enzyme interaction property suggests that the compound may cause drug-drug interactions. The cumulative clearance value can be anywhere from −1 to 2. Higher values signify a faster clearance, impacting how often a dose is given and how long the effect lasts. Compounds identified as substrates of Renal Organic Cation Transporter 2 (OCT2) are likely to be excreted via renal pathways mediated by this transporter. Should the compounds exhibit no interaction, this suggests that there are alternative routes for renal excretion.

Based on the analysis from the pkCSM webserver, it was observed that several compounds, including Norwogonin, Wogonin, Butein, Tricin, Eupatilin, Nevadensin, and 6-Methoxytricin, demonstrated moderate to high intestinal absorption (ranging from 75% to over 93%) and acceptable water solubility profiles, indicating favorable oral bioavailability. These compounds also exhibited adequate permeability in the Caco-2 model and were largely non-inhibitors of P-glycoprotein, reducing the risk of efflux-related bioavailability limitations. Furthermore, most of these ligands showed limited blood-brain barrier (BBB) permeability and moderate clearance rates, suggesting suitability for peripheral therapeutic targets without extensive CNS exposure.

Conversely, compounds such as Nobiletin, 5-Hydroxy-3,7,8,3′,4′-pentamethoxyflavone, 3,5,6,7,8-Pentamethoxyflavone and 17-DMAG, although demonstrating high intestinal absorption, presented less favourable pharmacokinetic profiles due to their significant interaction with metabolic enzymes (notably as CYP3A4 substrates and inhibitors), potential P-glycoprotein inhibition, and in some cases lower predicted clearance. Levodopa, despite its clinical relevance, showed low intestinal absorption and poor permeability, which aligns with its known pharmacokinetic limitations.

**Table 3:** Extensive ADMET profiling with pkCSM software.; VDss: The Volume of distribution at steady state; Caco2: Carcinoma colon-2 cells permeability of the CNS: permeability of the Central Nervous System; Renal OCT2: Organic cation transporter 2 in the kidneys; CYP2D6: CYP3A4: Cytochrome P450 3A4; Cytochrome P450 2D6.

**LIGANDS**	**Water solubility (log mol/L)**	**Caco2 permeability (log Papp in 10–6 cm/s)**	**Intestinal absorption (human) (% Absorbed)**	**Skin Permeability (log Kp)**	**P-glycoprotein I inhibitor**	**P-glycoprotein II inhibitor**	**VDss (human) (log L/kg)**	**BBB permeability (log BB)**	**CNS permeability (log PS)**	**CYP2D6 substrate**	**CYP3A4 substrate**	**CYP2D6 inhibitor**	**CYP3A4 inhibitor**	**Total Clearance (log ml/min/kg)**	**Renal OCT2 substrate**
Levodopa	-0.55	-0.397	46.188	-3.065	No	No	-0.329	-0.752	-3.036	No	No	No	No	0.446	No
Nobiletin	-5.139	1.117	98.773	-3.111	Yes	Yes	-1.065	-0.946	-2.939	No	Yes	No	Yes	0.69	No
Nevadensin	-4.218	0.388	87.069	-3.422	Yes	No	-1.044	-0.711	-2.913	No	Yes	No	No	0.58	No
3,5,6,7,8-Pentamethoxyflavone	-4.97	1.101	98.776	-3.163	Yes	Yes	-0.941	-0.718	-2.807	No	Yes	No	Yes	0.247	No
Eupatilin	-4.218	0.388	87.069	-3.422	Yes	No	-1.044	-0.711	-2.913	No	Yes	No	No	0.605	No
Butein	-2.994	0.37	75.112	-3.476	No	No	-0.732	-0.836	-2.389	No	No	No	No	0.047	No
Norwogonin	-3.299	1.062	90.14	-3.433	No	No	-0.824	-0.708	-2.059	No	No	No	No	0.276	No
Tricin	-3.86	0.284	82.414	-3.473	No	No	-1.083	-1.106	-2.965	No	Yes	No	No	0.606	No
Wogonin	-3.691	1.064	93.021	-3.331	No	No	-0.785	-0.23	-2.033	No	No	No	No	0.32	No
17-DMAG	-4.292	-0.202	47.569	-2.863	Yes	No	-0.119	-1.481	-3.76	No	Yes	No	No	-0.382	No
5-Hydroxy-3,7,8,3′,4′-pentamethoxyflavone	-4.807	0.401	90.091	-3.237	Yes	Yes	-1.122	-0.943	-2.992	No	Yes	No	Yes	0.643	No
6-Methoxytricin	-4.126	0.194	80.782	-3.373	No	No	-1.209	-1.305	-3.097	No	Yes	No	No	0.568	No

### 3.2 Searching MFs-associated targets

An initial set of 1000 target genes was identified utilizing the Swiss Target Prediction database (refer to the supplementary file S2). Duplicate entries were afterward removed using Venny 2.1.0 tool (https://bioinfogp.cnb.csic.es/tools/venny/), resulting in a refined list of 172 putative target genes associated with the 10 MFs. On average, every compound was estimated to engage with around 100 possible targets (refer to the supplementary file S2). This indicates a broad and overlapping pharmacological profile, emphasizing methoxy flavonoids' multi-target activity.

### 3.3 Obtaining targets related to MFs in Parkinson's disease

**Fig. 3:**
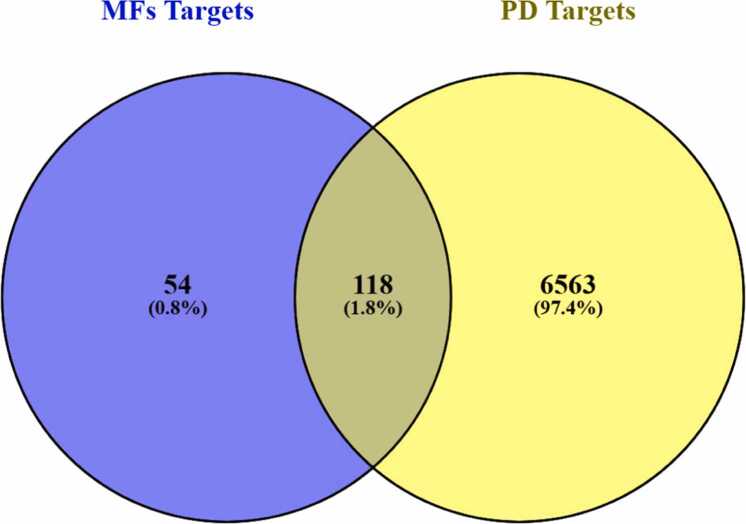
Gene Targets related to methoxy flavonoids in PD (Venny 2.1.0).

After identifying potential drug targets, we retrieved a total of 6681 genes linked to PD out of the GeneCards database (see supplementary file S3). Subsequently, Overlapping targets between MF-related genes were identified through Venn diagram analysis and genes associated with PD (illustrated in Fig. 3). This analysis revealed 118 common genes, which were considered putative therapeutic targets and designated as hub genes potentially involved in neuroprotection against PD.

### 3.4 Protein-protein interaction network analysis and node screening

**Fig. 4:**
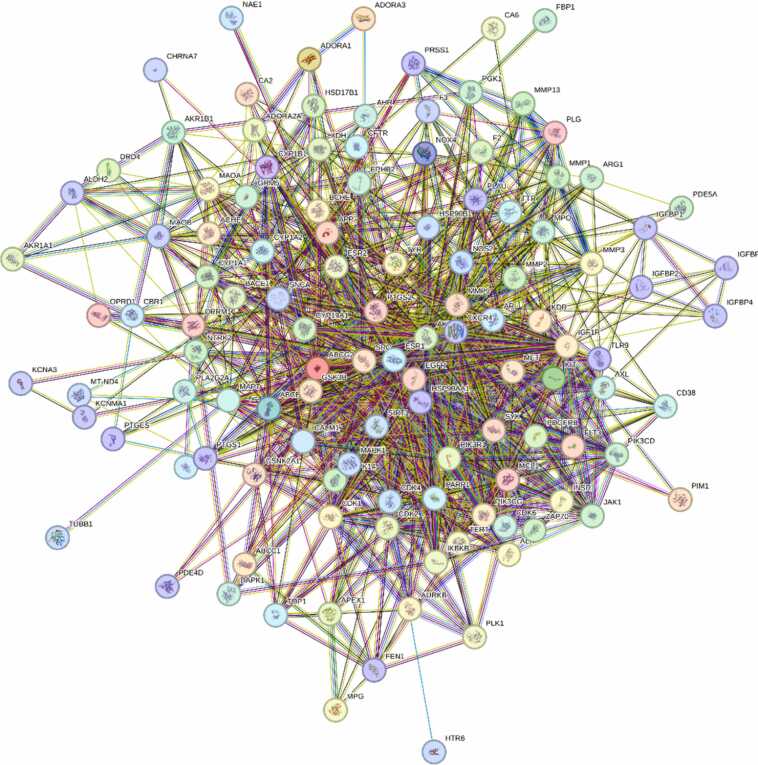
STRING database (version 12.0) was employed to generate the PPI network between MFs and PD-associated targets.

**Fig. 5:**
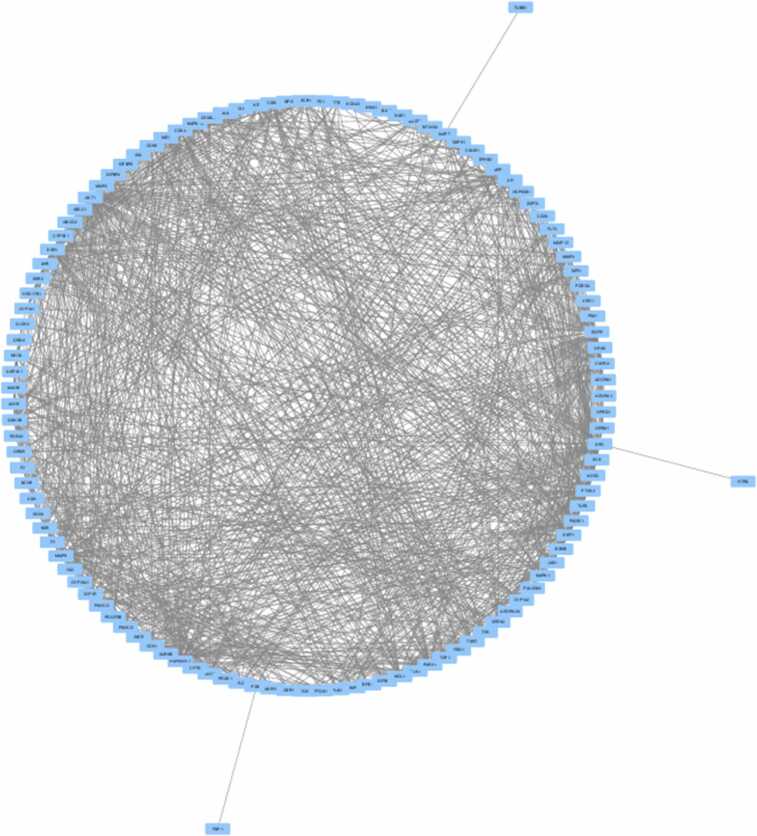
Network construction of hub-genes using Cytoscape.

**Fig. 6:**
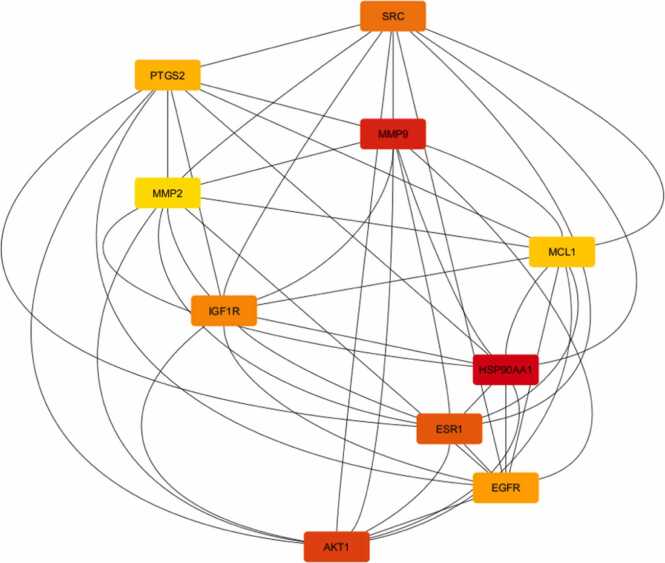
Network evaluation of highly ranked targets. The red colour signifies the highest level of interaction (highly interactive), orange indicates a moderate level (moderate interactive), and yellow denotes a mild level (mild interactive).

The network comprised 118 nodes and 1096 edges (Fig. 4, Fig. 5). As illustrated in Fig. 5, the constructed network provides a comprehensive visualization of the predicted interactions. To quantify the topological importance of these nodes within this specific network, an analysis of node degree (edge counts) was conducted. The most highly modulated targets, characterized by the greatest number of direct connections, include AKT1 (degree = 73), EGFR (degree = 68), SRC (degree = 65), ESR1 (degree = 64), HSP90AA1 (degree = 59) and MMP9 (degree = 55). The high edge counts of these specific nodes indicate their central topological role in mediating the complex multi-target interactions of the methoxylated flavonoids prior to the strict MCC hub-gene screening. Key network metrics, including a mean node degree of 18.6, a network diameter of 4, and a network density of 0.159, indicated a highly interconnected system. Among these, the top hub genes HSP90AA1, MMP9, AKT1, ESR1, SRC, IGF1R, EGFR, PTGS2, MCL1, and MMP2 were identified (Fig. 6) due to their significantly elevated scores (refer to the supplementary file S4). Key network metrics, such as displaying a mean node degree of 18.6 and a compact network diameter, indicated a highly interconnected system. These findings suggest strong functional associations among the targets, supporting their potential involvement in Parkinson’s disease pathogenesis.

### 3.5 Construction of compound-target-pathway network

**Fig. 7:**
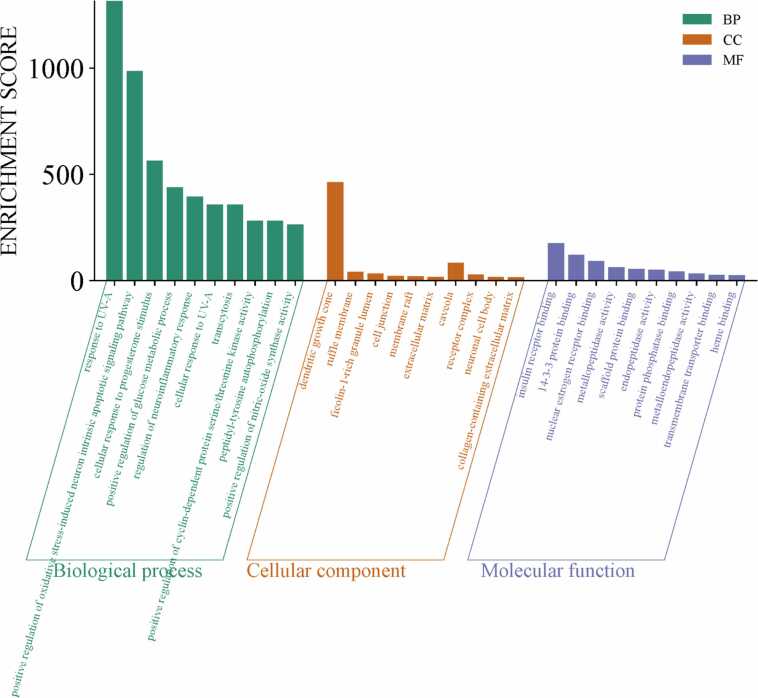
Pathways of the hub-genes (Biological process, Cellular Component, Molecular Function).

**Fig. 8:**
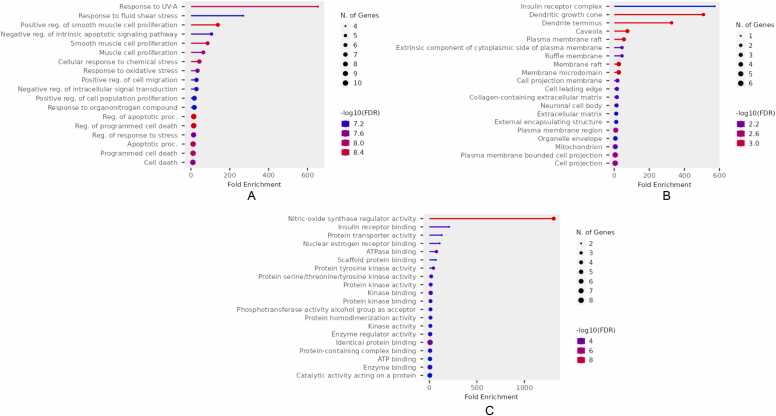
**A.** Gene ontology plots for Biological Process. **B.** Gene ontology plots for Cellular components. **C.** Gene ontology plots for Molecular functions.

The DAVID database was utilized for functional annotation and pathway enrichment of the selected targets. GO enrichment analysis classified the targets into three major categories: Cellular Components (CC), Molecular Functions (MF) and Biological Processes (BP) (Fig. 7). The analysis revealed that the majority of the methoxy flavonoid targets were involved in key neuroprotective processes, including oxidative stress response, apoptotic regulation, and synaptic plasticity. In the CC category, enriched terms included plasma membrane components, dendritic growth cones, and extracellular matrix structures, highlighting roles in neuronal connectivity and repair. The MF category was dominated by receptor binding (insulin and oestrogen), metal ion binding, and transmembrane receptor activity, indicating involvement in signal transduction and redox homeostasis (refer to Fig. 8A-C and Supplementary File S5). These enriched pathways and molecular functions suggest that MFs may exert their therapeutic impacts in PD by modulating oxidative stress, promoting neuronal survival, and supporting synaptic function.

### 3.6 KEGG (Kyoto Encyclopedia of Genes and Genomes) enrichment analysis

This analysis uncovered that MFs might provide neuroprotection in PD by influencing the PI3K-Akt signaling pathway, which is crucial for controlling cell survival, apoptosis, and oxidative stress. Methoxy flavonoids were predicted to interact with key signalling proteins including mTOR (mammalian target of rapamycin), PI3K (phosphoinositide 3-kinase), GSK-3β, BAD (Bcl-2-associated death promoter) and Akt (protein kinase B). These targets collectively contribute to dopaminergic neuronal survival, neuroprotection, and the regulation of inflammation and neurotransmitter homeostasis.

**Fig. 9:**
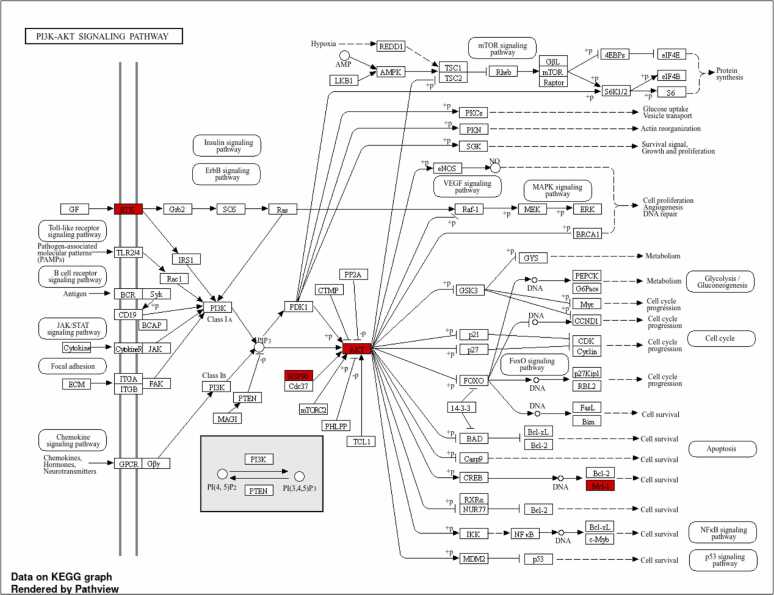

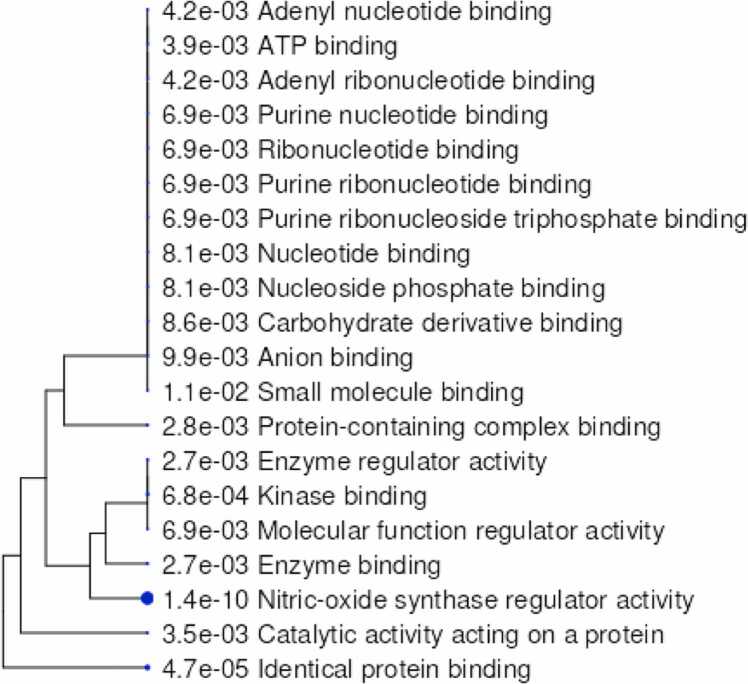

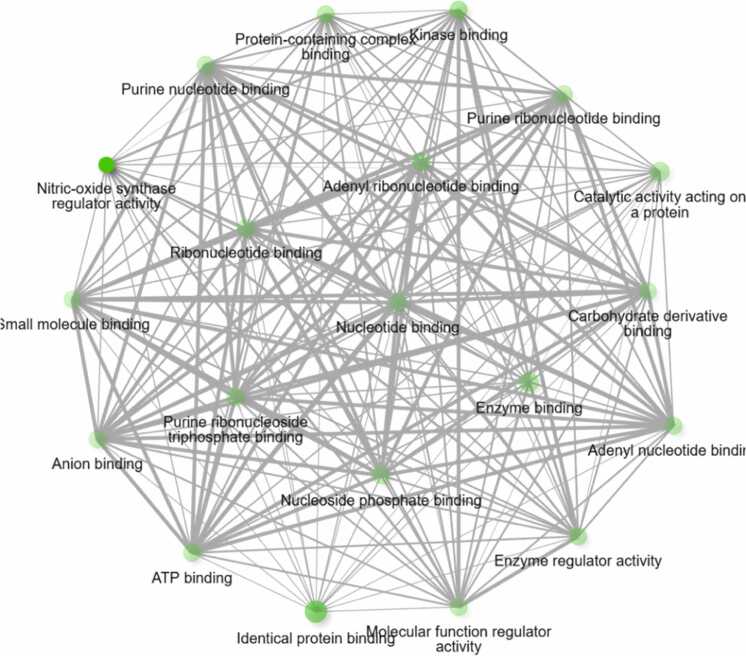
**A.** KEGG Pathway (PI3K-Akt signaling pathway, map04151). Permission to reproduce this image was granted by Kanehisa Laboratories. (Source: The pathway map was generated using the KEGG pathway database. The image is reproduced with permission from Kanehisa Laboratories [50], [52]. **B.** Targeted genes Enrichment analysis: Clustering between pathways. **C.** Enrichment analysis of targeted genes: Network analysis.

Specifically, PI3K activates Akt signalling, promoting neuronal survival and inhibiting apoptosis, while Akt itself regulates essential cellular processes such as growth, metabolism, and apoptosis. mTOR is crucial in stress response modulation, neuroprotection, protein synthesis and supporting neuronal health under degenerative conditions. GSK-3β is a key modulator of neuronal apoptosis and survival pathways, with critical roles in neurodegeneration. Meanwhile, BAD functions as a pro-apoptotic regulator involved in neuronal cell death processes (Fig. 9A).

To summarize the relationships between enriched pathways, a hierarchical clustering tree was constructed, grouping together pathways that had a high number of overlapping genes in common. In the visual representation, larger dots indicate pathways with greater statistical significance (lower P-values) (Fig. 9B). To further illustrate these relationships, an interaction network was generated, where nodes represent individual pathways. In this network, larger nodes correspond to larger gene sets, darker shades reflect higher enrichment scores, and thicker borders indicate greater gene overlap between pathways (Fig. 9C).

### 3.7 Molecular docking

**Fig. 10:**
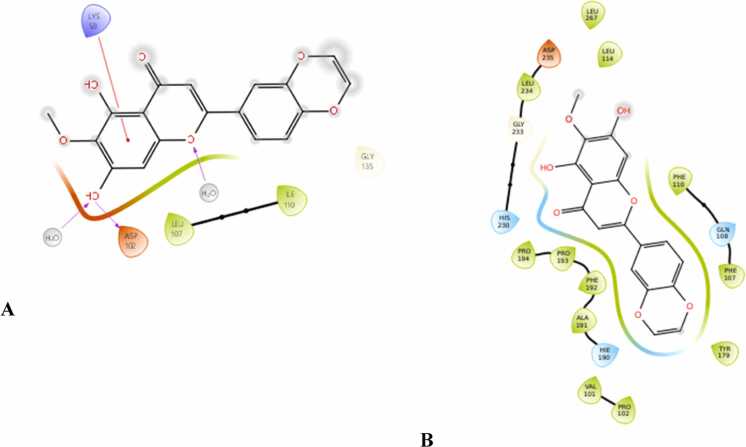
2-Dimensional molecular docking image of Eupatilin with a) PDB: 7LT0 and b) PDB: 8K5Y.

**Fig. 11:**
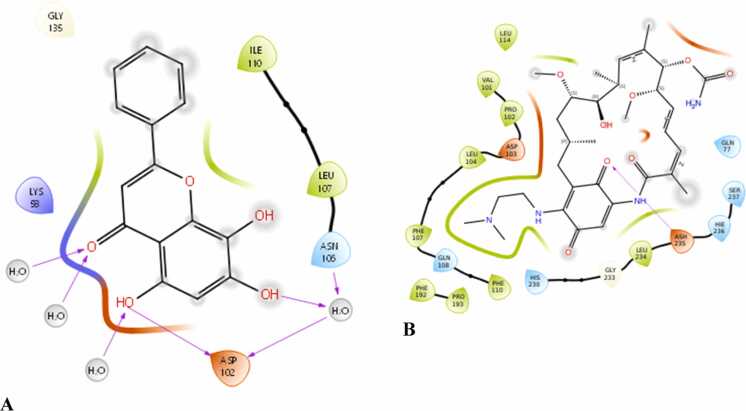
2-Dimensional molecular docking image of Norwogonin with a) PDB: 7LT0 and b) PDB: 8K5Y.

Molecular docking analysis was carried out to investigate the interactions of selected flavonoids with the two target proteins: 7LT0 and 8K5Y. The binding energies (in kcal/mol) of the active compounds are summarised in Table 4. Among the screened ligands, Eupatilin (Fig. 10) and Norwogonin (Fig. 11) exhibited the strongest binding interactions across both protein targets. Norwogonin showed the best binding affinity for 8K5Y (–10 kcal/mol) and maintained a good interaction with 7LT0 (–4.7 kcal/mol). Conversely, Eupatilin achieved the highest binding energy against 7LT0 (–6.3 kcal/mol) and a robust interaction with 8K5Y (–9.7 kcal/mol). Other notable candidates include Wogonin, Butein and Tricin, showcasing stable binding energies of −9.4 to −9.2 kcal/mol with 8K5Y and −4.3 to −5 kcal/mol with 7LT0. Nevadensin showed significant affinities, with values of −4.3 kcal/mol for 7LT0 and −8.9 kcal/mol for 8K5Y. The reference ligands, levodopa and 17-DMAG, exhibited binding energies spanning from 7.4 to –6.7 kcal/mol indicating that the tested MFs exhibited comparable or superior binding affinities. Overall, the docking results imply that the MFs could establish a stable interaction with both 7LT0 and 8K5Y, which may contribute to their biological activity.

**Table 4:** Docking results of active compound and target protein.

**Ligand**	**Binding energy (kcal mol**^**−1**^**)**	
	**PDB: 7LT0**	**PDB: 8K5Y**
Eupatilin	-6.3	-9.7
Norwogonin	-4.7	-10
Butein	-5	-9.2
Levodopa	-4.8	-6.7
Tricin	-4.4	-9.2
6-Methoxytricin	-4.3	-8.7
Nevadensin	-4.3	-8.9
Wogonin	-4.3	-9.4
Nobiletin	-3.6	-7.6
5-Hydroxy-3,7,8,3′,4′-pentamethoxyflavone	-3.2	-8.6
3,5,6,7,8-Pentamethoxyflavone	-2.8	-7.2
17-DMAG	7.4	-5

### 3.8 Molecular dynamics

**Fig. 12:**
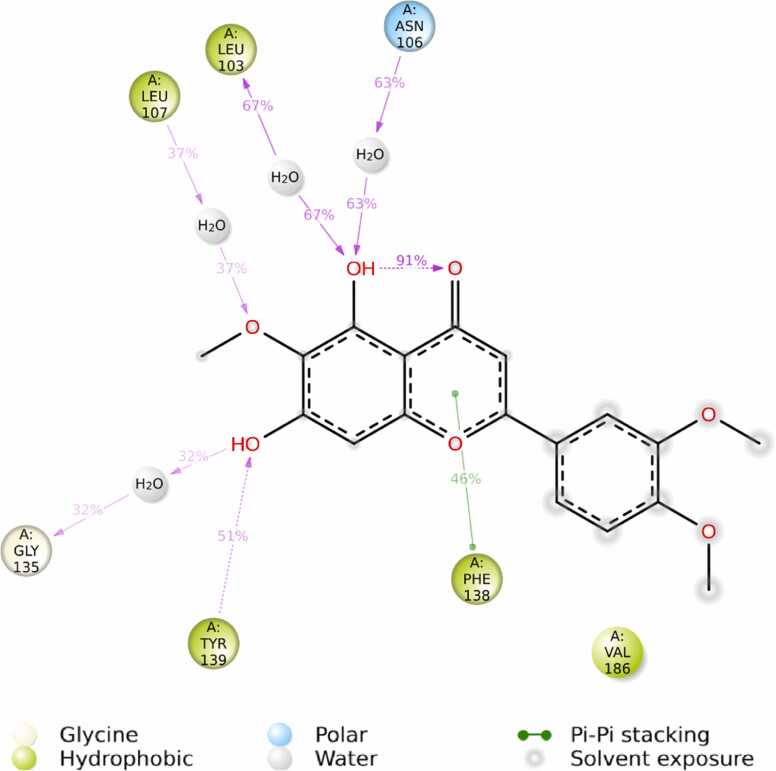
Ligand-protein contact between Eupatilin and 7LT0.

**Fig. 13:**
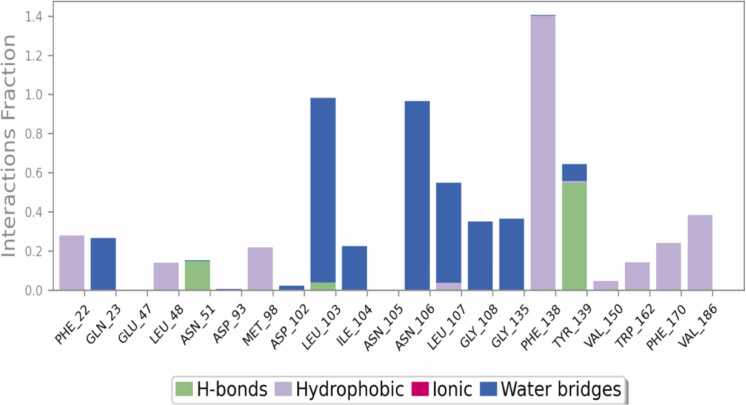
Analysis of hydrogen bonds after a 100 ns molecular dynamics simulation. The crucial hydrogen bonds formed by Eupatilin with the amino acid residues ASN51 and TYR139 of 7LT0 are shown. Such interactions are crucial for stabilizing the ligand–target complex.

**Fig. 14:**
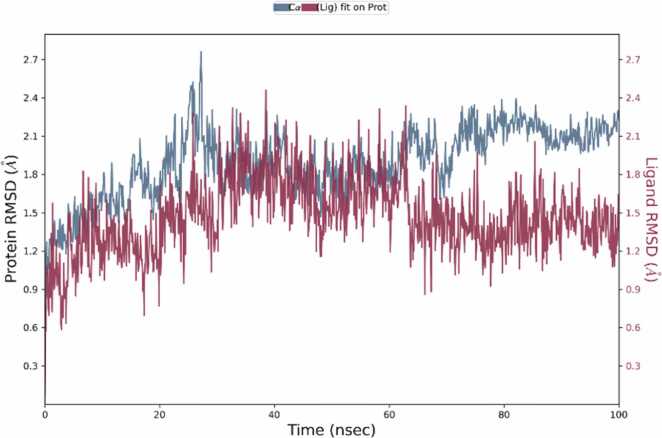
RMSD plot of the Eupatilin-7LT0 complex over 100 ns of molecular dynamics simulation. Eupatilin is shown in the red colour and 7LT0 in the blue colour.

**Fig. 15:**
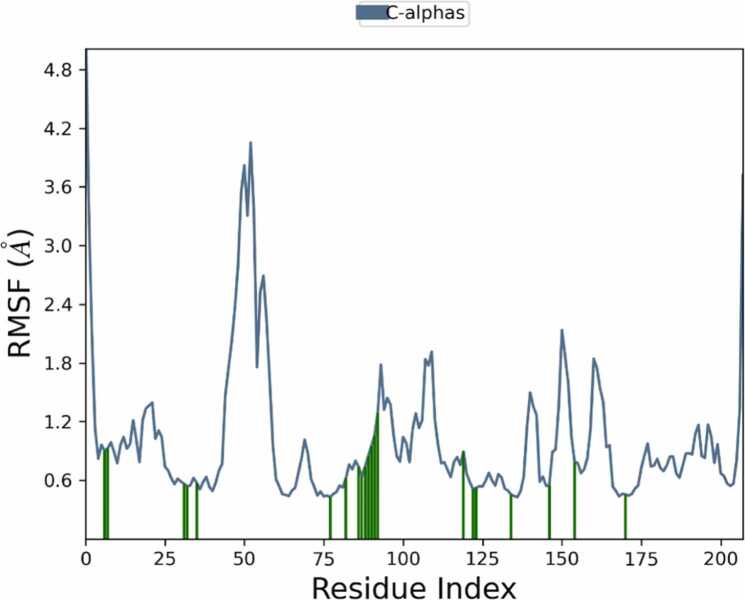
RMSF plot of the Eupatilin-7LT0 complex during 100 ns of molecular dynamics simulation.

Eupatilin exhibited the most robust binding interaction with 7LT0 in the docking studies among all the ligands, resulting in its selection for MD simulations. A 100 ns MD simulation was performed to evaluate the dynamic stability and conformational behavior of the Eupatilin–7LT0 protein complex. The 2D interaction diagram (Fig. 12) revealed that Eupatilin maintained stable hydrogen interacts with crucial residues like ASN106, LEU103, LEU107, GLY135, TYR139, and PHE138, along with several water-mediated interactions. Hydrophobic and polar interactions contributed to the overall binding stability, with interaction occupancy percentages indicating persistent contact throughout the simulation. The protein–ligand contact histogram (Fig. 13) showed that residues LEU103, PHE138, TYR139, and ASN106 exhibited the highest interaction frequencies, supporting the strong binding affinity observed in docking studies. This suggests that Eupatilin maintained stable interactions with these residues during the simulation trajectory. The RMSD plot (Fig. 14) demonstrated that the protein backbone (Cα atoms) stabilized around 2.0 Å to 2.5 Å, indicating overall conformational stability. The ligand RMSD remained stable within the 1.0 Å to 1.8 Å range, suggesting that Eupatilin maintained a consistent binding pose inside the active site during the entire simulation. RMSF analysis (Fig. 15) indicated minimal fluctuations in most protein residues, with higher flexibility observed around loop regions and terminal residues. Notably, residues involved in ligand binding, such as LEU103 and ASN106, showed low fluctuation, reflecting stable interactions with Eupatilin. Overall, the molecular dynamics simulation confirmed that Eupatilin formed a stable complex with the 7LT0 protein, maintaining key interactions and demonstrating minimal conformational fluctuations over the 100 ns trajectory, supporting its potential as a neuroprotective candidate in Parkinson’s disease.

## 4 Discussion

Parkinson’s disease, which James Parkinson first characterized in 1817, is the second most frequent neurodegenerative disease and presents a major worldwide health challenge. Motor symptoms, such as tremors, rigidity, bradykinesia and postural instability, arise from the progressive degeneration of dopaminergic neurons in the substantia nigra along with non-motor features like cognitive decline, mood disturbances, and sleep disorders. Despite advances in symptomatic therapies including levodopa-carbidopa combinations and MAO-B inhibitors, no disease-modifying treatments are currently available [58], [59]. Furthermore, long-term use of existing drugs often leads to adverse effects, highlighting the urgent need for safer, multi-target therapies that address the complex pathogenesis of PD.

This research utilized a molecular dynamics, molecular docking and network pharmacology-based methodology to investigate the multi-target therapeutic potential of methoxylated flavonoids (MFs) in PD. ADMET and Drug-likeness profiling of 10 selected MFs revealed favorable pharmacokinetic and safety profiles. All compounds passed the Lipinski and Ghose filters, supporting their oral bioavailability and drug-like physicochemical properties [60]. SwissADME analyses indicated promising gastrointestinal absorption, and notably, 3,5,6,7,8-pentamethoxyflavone showed potential blood-brain barrier (BBB) permeability, a key criterion for CNS-targeted therapeutics [61]. Toxicity assessments through ProTox 3.0 and pkCSM predicted high LD50 values and minimal risks of hepatotoxicity or neurotoxicity, aligning with previous reports on the safety of natural polyphenols [62], [63].

Although variations in BBB permeability and CNS penetration were observed across the compounds, flavonoids like Eupatilin, Nobiletin, and Wogonin demonstrated favorable properties, reinforcing their candidacy for neuroprotective roles in PD [64]. Collectively, these ADMET findings established a solid pharmacological foundation, justifying the compounds' selection for mechanistic exploration.

Network pharmacology revealed that the selected MFs target a broad array of PD-associated pathways. Of 1000 putative flavonoid targets, 172 were identified after duplicate removal, with 118 overlapping with 6681 PD-associated genes. The protein–protein interaction network, built using STRING and displayed with Cytoscape, showed a highly interconnected structure comprising 118 nodes and 1096 edges. Maximal Clique Centrality (MCC) analysis identified hub proteins including HSP90AA1, MMP9, AKT1, ESR1, SRC, IGF1R, EGFR, PTGS2, MCL1, and MMP2, all central regulators of apoptosis, neuroinflammation, oxidative stress, and synaptic function [65], [66], [67], [68], [69], [70], [71], [72], [73], [74], [75], [76].

Gene Ontology (GO) enrichment highlighted Biological Processes like oxidative apoptosis regulation, stress response and synaptic plasticity which are critical pathways disrupted in PD. Cellular Components enrichment included dendritic growth cones, plasma membranes, and extracellular matrix structures essential for neuronal function and repair. Molecular Functions emphasized receptor binding (insulin, oestrogen), metal ion binding, and transmembrane receptor activity, reflecting their roles in neuronal signaling and homeostasis.

KEGG pathway enrichment analysis highlighted the PI3K-Akt signaling pathway. which is a crucial survival axis for dopaminergic neurons. The study predicted interactions with major regulators including PI3K, Akt, mTOR, BAD, and GSK-3β. Dysregulation of this pathway particularly GSK-3β overactivation and mTOR dysfunction is strongly associated with dopaminergic neuron apoptosis and neurodegeneration in PD. The ability of methoxylated flavonoids to simultaneously modulate these multiple nodes highlights their potential to overcome the limitations of single-target therapies. [77], [78], [79].

Hierarchical clustering and pathway network analyses demonstrated that the enriched pathways shared substantial gene overlap, suggesting coordinated modulation of interlinked biological modules. This systems-level modulation by MFs addresses the multifactorial nature of PD pathology, including oxidative stress, neuroinflammation, and apoptotic signaling.

Studies using molecular docking confirmed the interaction of MFs with essential PD targets, HSP90AA1 (PDB: 7LT0) and MMP9 (PDB: 8K5Y). Eupatilin and Norwogonin emerged as the top ligands, exhibiting binding energies comparable to or exceeding those of reference compounds Levodopa and 17-DMAG. Eupatilin showed the strongest binding affinity for 7LT0 (–6.3 kcal/mol), while Norwogonin displayed robust interaction with 8K5Y (–10 kcal/mol). Docking analyses revealed that Eupatilin interacted with critical residues such as ASN106, LEU103, and TYR139, residues implicated in stabilizing HSP90AA1’s functional conformation [80], [81].

It is worth mentioning that HSP90AA1 is crucial for PD because it stabilizes misfolded proteins and modulating proteostasis. The aggregation of α-synuclein, a characteristic of PD pathology, is accelerated by the dysregulation of HSP90, which makes it a prospective target for therapy [82], [83]. Similarly, MMP9 helps to blood-brain barrier disruption and neuroinflammation in PD, further exacerbating neurodegeneration [84], [85]. Modulating these targets, alongside PI3K-Akt-mTOR signaling and GSK-3β inhibition, offers a comprehensive strategy to protect dopaminergic neurons from multiple pathological insults.

In PD models, natural substances like Safflower flavonoid and Apigenin have demonstrated neuroprotective effects, as well as Fisetin. Safflower flavonoid extract protects against PD by reducing neuroinflammation through NLRP3 inflammasome inhibition and promoting the survival of dopaminergic neurons [86]. By altering gut microbiota composition and diversity, fisetin helps protect neurons from neurodegeneration [87]. In C57BL/6 mice, the administration of apigenin reduced the histopathological changes caused by MPTP in brain tissue by reversing the changes in expressions and levels of TNF-α, IL-1β, IL-6, IL-10, and TGF-β [88]. In contrast to these compounds, methoxylated flavonoids such as Eupatilin and Norwogonin demonstrate broader multi-target effects by concurrently modulating oxidative stress, protein aggregation, apoptosis, and neuroinflammatory pathways.

The dynamic stability of the Eupatilin–7LT0 complex over a 100 ns trajectory was further confirmed through molecular dynamics (MD) simulations. The RMSF and RMSD analyses showed that the ligand preserved a stable binding pose with minimal conformational fluctuations. Persistent hydrophobic interactions and hydrogen bonding with key residues contributed to the complex's overall structural stability. These results corroborate with prior reports on Eupatilin’s neuroprotective effects in apoptosis and models oxidative stress [89], [90].

Despite the promising neuroprotective properties of methoxylated flavonoids, their clinical application is limited by poor water solubility, rapid first-pass metabolism, and restricted permeability across the blood–brain barrier (BBB). Many sophisticated drug delivery strategies have been investigated to tackle these pharmacokinetic challenges [91].

This research introduces a new integrative strategy for tackling the difficulties in treating Parkinson’s disease by using network pharmacology, molecular dynamics simulations and molecular docking to explore the multi-target potential of methoxylated flavonoids. This study underscores the ability of methoxy flavonoids to modulate multiple PD-relevant targets, such as HSP90AA1, MMP9, AKT1, GSK-3β, and mTOR, in contrast to traditional PD treatments that usually aim to restore dopaminergic activity or inhibit a single target.

Eupatilin was identified as a lead compound among the tested flavonoids, exhibiting the strongest binding affinity and stable interaction with HSP90AA1, corroborated by molecular dynamics simulations. Eupatilin's interaction with HSP90AA1, a chaperone that plays a role in protein homeostasis and α-synuclein aggregation, is particularly noteworthy, as it offers a unique therapeutic mechanism that has not been thoroughly investigated in relation to PD. Eupatilin shows potential as a multi-target agent that can diminish neuroinflammation, avert protein misfolding, and promote neuronal survival by focusing on this crucial pathway. This study is among the first to thoroughly clarify how methoxy flavonoids interact with HSP90AA1 in PD, highlighting Eupatilin as a potential candidate for disease modification that goes beyond merely alleviating symptoms. The present molecular dynamics simulations concentrated on the Eupatilin–HSP90AA1 complex because of its robust binding affinity and advantageous pharmacokinetic profile. This selective focus was chosen to allow for a detailed mechanistic assessment while using resources efficiently.

Eupatilin demonstrated a broader pharmacological profile compared to levodopa, which is the standard symptomatic therapy for PD that restores dopamine levels but does not alter disease progression. Eupatilin can influence several pathological processes in PD, such as oxidative stress, apoptosis, protein aggregation, and neuroinflammation, by targeting HSP90AA1, MMP9, AKT1, and GSK-3β at the same time. This suggests that it may have disease-modifying effects rather than serving solely to manage symptoms. HSP90AA1 is vital to the advancement of PD because it stabilizes client proteins that contribute to neurodegeneration. Meanwhile, MMP9 and GSK-3β play roles in neuroinflammation and apoptotic pathways, respectively. Eupatilin's stable binding to HSP90AA1 indicates its potential therapeutic significance in targeting proteostasis mechanisms, which are gaining recognition in clinical research as viable intervention points beyond dopamine-centric strategies.

It is recognized, though, that simulating further complexes of flavonoids and targets, like Norwogonin with MMP9 or AKT1, could enhance these mechanistic understandings. Future research will seek to include wider simulations in order to improve the translational significance of the results. Moreover, a comparative examination of Eupatilin's binding profile with established HSP90AA1 inhibitors like 17-DMAG would offer additional insight into its inhibitory potential. Although our research concentrated on methoxy flavonoids that are not well-studied, we did not conduct a distinct molecular dynamics simulation of the apo-form of HSP90AA1. Future studies could include such simulations to enhance understanding of conformational dynamics induced by ligands. Future studies should focus on experimental and preclinical validation, investigate advanced strategies for nanocarrier delivery to improve brain bioavailability, and evaluate combination therapies in order to fully harness the neuroprotective potential of methoxy flavonoids in Parkinson’s disease.

## 5 Conclusion

This research offers an extensive *in silico* examination of methoxylated flavonoids as potential multi-target neuroprotective agents for Parkinson's disease. Using integrative network pharmacology, molecular docking, and molecular dynamics simulations, we pinpointed essential targets such as HSP90AA1 and MMP9 that play crucial roles in regulating neuronal survival, oxidative stress, and apoptosis. Methoxy flavonoids, especially Eupatilin, showed a stable interactions and strong binding affinity with these targets, supporting their potential role in modulating multiple pathogenic pathways associated with PD. Given the emerging role of HSP90AA1 and MMP9 in PD pathogenesis, the stable interaction of Eupatilin with these proteins warrants further investigation. The study highlights the therapeutic promise of flavonoids as modulators of the PI3K-Akt signaling axis and proteostasis regulators, offering a holistic approach beyond conventional dopaminergic therapies. To convert these computational insights into workable therapeutic strategies for modifying Parkinson’s disease, future experimental studies, both *in vitro* and *in vivo*, as well as those involving enhanced drug delivery systems are warranted.

## Limitation

This research was carried out as an *in silico* analysis to evaluate the multi-target therapeutic potential of flavonoids concerning Parkinson’s disease. This stage did not include any experimental validation nor did it employ advanced end-state free energy calculations, such as MM-GBSA to quantitatively validate the docking affinities. Recognizing the limitations of computational predictions, we propose future MM-GBSA evaluations be conducted alongside rigorous experimental pipelines. Future studies will evaluate Eupatilin utilizing SH-SY5Y cellular models of Parkinson's-like pathology to assess neuroprotection using MTT assays, and *in vivo* validation utilizing a zebrafish model to observe behavioural and neurobehavioral recovery. To explicitly confirm the predicted multi-target mechanism, western blotting will be utilized to quantify the modulation of the primary identified hub proteins, particularly HSP90AA1 and MMP9. These forthcoming experimental studies will be essential for translating our computational findings into clinically relevant therapeutic strategies.

## Ethics approval and consent to participate

This research did not include animal subjects or human participants. All analyses were conducted using datasets and computational tools that are publicly available. Consequently, ethical approval and consent to participate were unnecessary.

## Funding

The project has been funded under GATE - Young Faculty Research Grant (14/PROVC/2024), Sri Ramachandra Medical College and Research Institute, Porur, Chennai for the year 2023–2024 and INTI International Research Fellowship Program (IIU/HR/JSP/SYA/20809/25), INTI University, Malaysia for the year 2026-2028.

## CRediT authorship contribution statement

**Chetan Ashok:** Writing – review & editing, Conceptualization. **Srikanth Jeyabalan:** Supervision, Conceptualization. **Parasuraman Pavadai:** Software, Methodology. **Vignesh M:** Writing – review & editing, Data curation. **Devaraja Dravida Pandiyan S:** Writing – review & editing, Methodology, Formal analysis. **Durga Mohan:** Writing – original draft, Software. **Revathi Sugumar:** Writing – review & editing, Data curation. **Ramya Sugumar:** Methodology, Formal analysis. **Sugin Lal Jabaris:** Formal analysis, Conceptualization. **Vetriselvan Subramaniyan:** Writing – review & editing, Methodology. **Ling Shing Wong:** Writing – review & editing, Methodology. **Mahendran Sekar:** Writing – review & editing, Methodology.

## Declaration of Generative AI and AI-assisted technologies in the writing process

During the preparation of this work, the authors used ChatGPT to correct spelling, grammar, and punctuation. After using this tool/service, the authors reviewed and edited the content as needed and took full responsibility for the publication’s content.

## Declaration of competing interest

The authors declare that they have no known competing financial interests or personal relationships that could have appeared to influence the work reported in this paper.

## Data Availability

Data will be made available on request.
