# Supplementary material for: Unravelling the multi-target mechanism of methoxylated flavonoids in Parkinson's disease: Insights from network pharmacology and molecular dynamics
Source: Toxicol Rep. 2026 Apr 1;16:102248. doi: 10.1016/j.toxrep.2026.102248 (PMC13091021; doi:10.1016/j.toxrep.2026.102248)
Supplement: Supplementary file 1 — Supplementary material [file mmc1.docx]

**Mechanistic insights into methoxylated flavonoids as potential neuroprotective agents: A network pharmacology, docking, and molecular dynamics approach**

**Supplementary File**

| 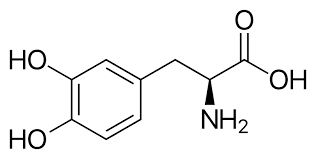 | 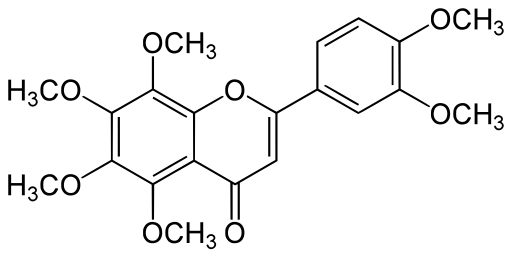 | 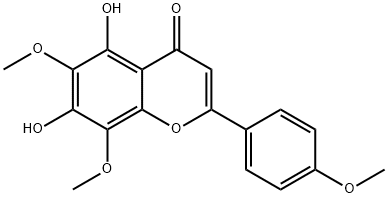 |
| --- | --- | --- |
| Levodopa | Nobiletin | 5,7-Dihydroxy-6,8,4'-trimethoxyflavone |
| 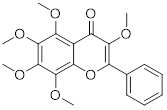 | 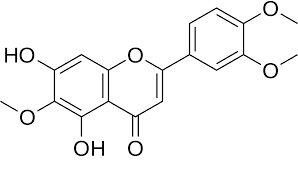 | 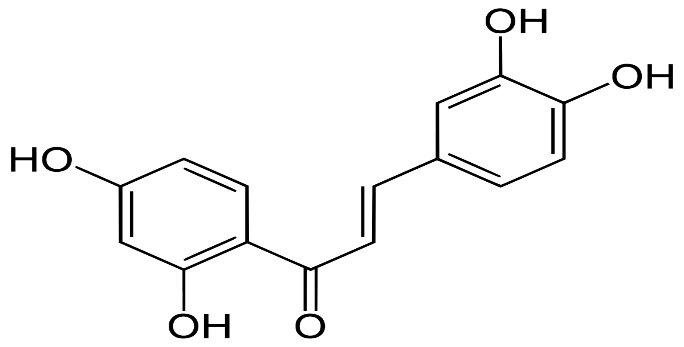 |
| 3,5,6,7,8-Pentamethoxyflavone | Eupatilin | Butein |
| 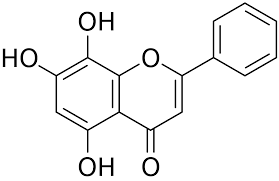 | 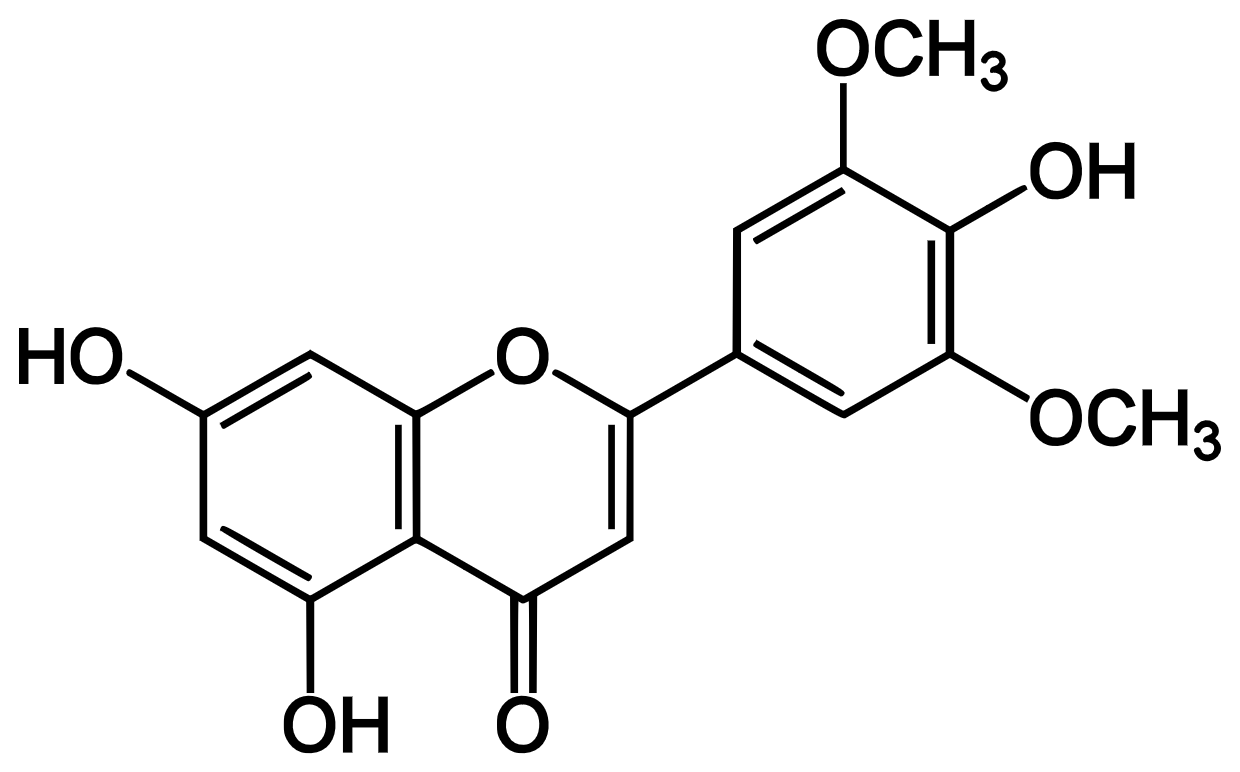 | 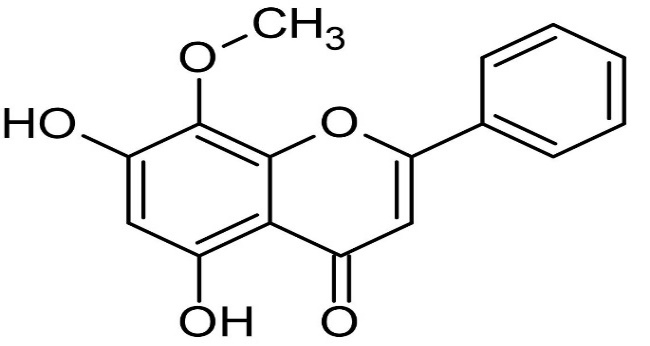 |
| Norwogonin | Tricin | Wogonin |
| 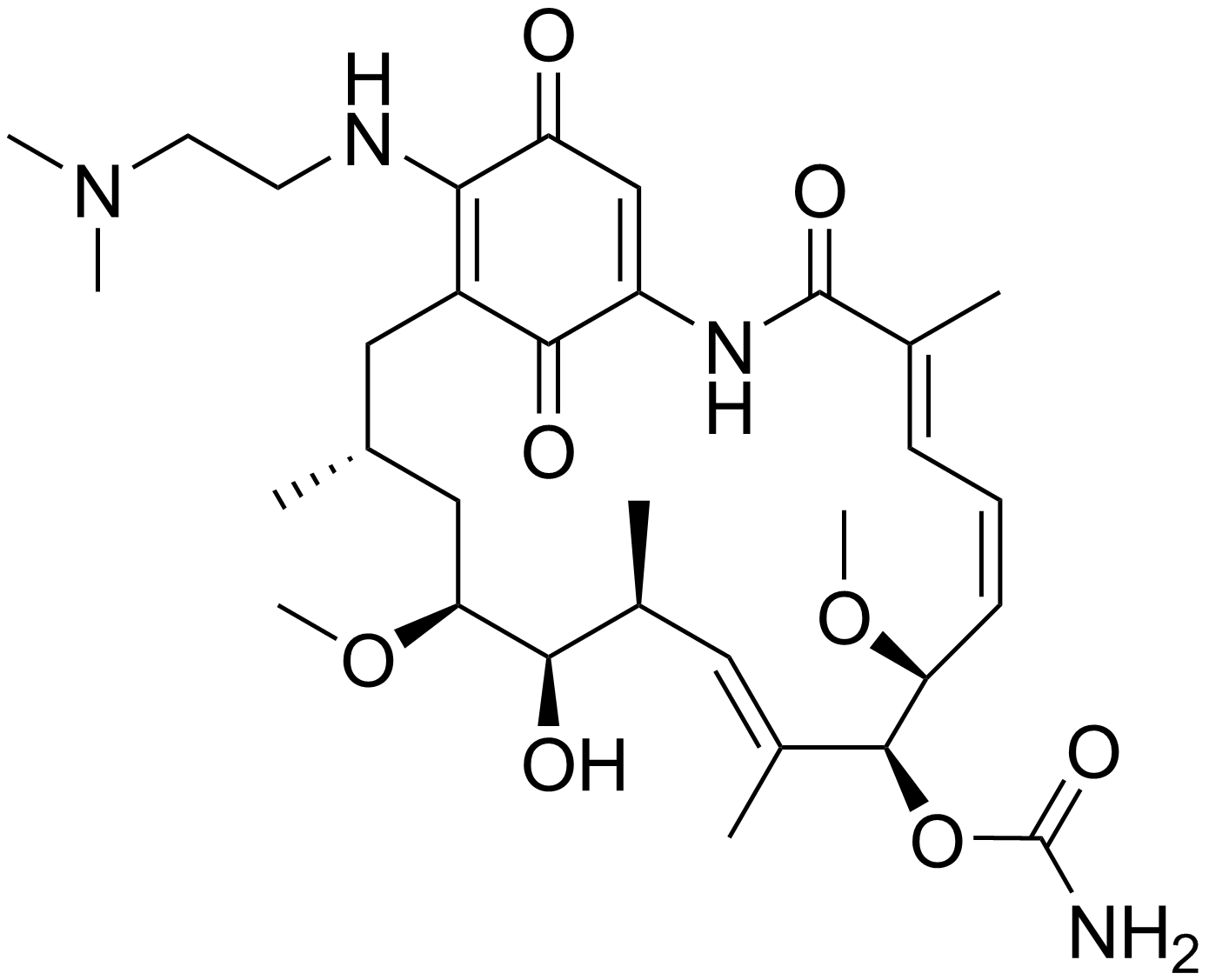 | 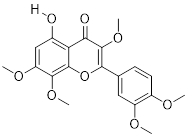 | 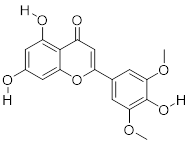 |
| 17-DMAG | 5-Hydroxy-3,7,8,3',4'-pentamethoxyflavone | 5,7,4'-Trihydroxy-6,3',5'-trimethoxyflavone |
